# Institutionalizing postpartum intrauterine device (IUD) services in Sri Lanka, Tanzania, and Nepal: study protocol for a cluster-randomized stepped-wedge trial

**DOI:** 10.1186/s12884-016-1160-0

**Published:** 2016-11-21

**Authors:** David Canning, Iqbal H. Shah, Erin Pearson, Elina Pradhan, Mahesh Karra, Leigh Senderowicz, Till Bärnighausen, Donna Spiegelman, Ana Langer

**Affiliations:** Department of Global Health and Population, Harvard T. H. Chan School of Public Health, Boston, USA

**Keywords:** Postpartum contraception, IUD, Impact evaluation

## Abstract

**Background:**

During the year following the birth of a child, 40% of women are estimated to have an unmet need for contraception. The copper IUD provides safe, effective, convenient, and long-term contraceptive protection that does not interfere with breastfeeding during the postpartum period. Postpartum IUD (PPIUD) insertion should be performed by a trained provider in the early postpartum period to reduce expulsion rates and complications, but these services are not widely available. The International Federation of Obstetricians and Gynecologists (FIGO) will implement an intervention that aims to institutionalize PPIUD training as a regular part of the OB/GYN training program and to integrate it as part of the standard practice at the time of delivery in intervention hospitals.

**Methods:**

This trial uses a cluster-randomized stepped wedge design to assess the causal effect of the FIGO intervention on the uptake and continued use of PPIUD and of the effect on subsequent pregnancy and birth. This trial also seeks to measure institutionalization of PPIUD services in study hospitals and diffusion of these services to other providers and health facilities. This study will also include a nested mixed-methods performance evaluation to describe intervention implementation.

**Discussion:**

This study will provide critical evidence on the causal effects of hospital-based PPIUD provision on contraceptive choices and reproductive health outcomes, as well as on the feasibility, acceptability and longer run institutional impacts in three low- and middle-income countries.

**Trial registration:**

Trial registered on March 11, 2016 with ClinicalTrials.gov, NCT02718222.

**Electronic supplementary material:**

The online version of this article (doi:10.1186/s12884-016-1160-0) contains supplementary material, which is available to authorized users.

## Background

Postpartum access to insertion of an intrauterine device (IUD) is critical for meeting women’s need for long-acting but reversible contraceptive protection. The IUD is safe, effective, long-acting, reversible, and convenient, especially when inserted in the immediate postpartum period [1]. The postpartum IUD (PPIUD) does not interfere with breastfeeding, is safe for use by all women, including HIV-positive women, is associated with less discomfort and fewer side effects than interval IUD insertion, and allows women to obtain safe, long-acting (up to 12 years), highly effective yet reversible contraceptive protection that can be made readily available to women delivering in health facilities. In countries where women do not return for postnatal follow-up visits because of cost, distance, or health system challenges PPIUD offers a convenient and cost-effective postpartum contraceptive option.

Despite these well-established benefits of PPIUD, its uptake continues to be low. During the year following the birth of a child, 40% of women are estimated to have an unmet need for contraception [2]. Fewer than 50% of women in 30 of the 43 countries with recent Demographic and Health Surveys (DHS) used any method of contraception during 9-11 months postpartum, and in 12 of these countries the use was below 20% [3]. The copper-bearing intrauterine device (copper IUD) is a safe and effective method that can be used immediately after delivery [4]; yet, only in three of the 43 counties (Egypt, Kyrgyz Republic and Tajikistan), were 20% or more of postpartum users of contraception relying on this method [3].

A number of studies and systematic reviews have evaluated the safety, efficacy and acceptability of immediate postpartum insertion of IUDs (within 10 min of delivery of the placenta) compared to delayed postpartum insertion (more than 10 min to 48 or 72 h following delivery) or interval insertion (after four or six weeks following delivery). Two systematic reviews of the evidence confirmed the safety and effectiveness of postpartum IUD insertions [1, 5]. Fifteen studies included in the first review showed that immediate IUD insertion was safe when compared with delayed postpartum and interval insertion. Immediate postpartum insertion had lower expulsion rates when compared to delayed postpartum insertion, but had higher expulsion rates when compared to interval insertion. The authors also noted that immediate insertion following Caesarean delivery demonstrated lower expulsion rates than immediate insertion following vaginal delivery. The review by Grimes et al. (2010) covered nine randomized controlled trials and showed higher expulsion rates of immediate postpartum IUD insertion than interval insertion [5]. Eroğlu et al. (2006) is the only study to compare immediate postpartum insertion and delayed postpartum IUD insertions with interval insertion [6]. Among 268 women using TCu 380A IUDs in one hospital in Ankara, Turkey, expulsions within a year after insertion were the highest in the group that had delayed IUD insertion (70%) as compared to immediate postpartum insertion (37%) and to those receiving interval insertion (7%; *p* < 0.003) [6].

Several studies reported acceptability of postpartum IUDs in diverse settings when information and counselling on postpartum contraceptive methods was provided to women [7–9]. To expand the use of PPIUD, high-quality counselling during the antenatal period and training of physicians and midwives is critical [10]. However, in countries where antenatal care rates are low or antenatal care does not include counseling on postpartum contraception due to lack of provider time or training, women may not receive information on PPIUD before coming to the hospital for delivery. Despite higher expulsion rates, providers may counsel these women after delivery and offer PPIUD insertion after the 10-min window for immediate PPIUD insertion has passed. Expulsions will be lower if: (a) the IUD is inserted within 10 min after delivery of the placenta; (b) placement is sufficiently high in the uterine fundus; and (c) insertion is done by a specially trained provider [1, 11, 12].

This trial is an impact evaluation of an intervention implemented by the International Federation of Gynaecology and Obstetrics (FIGO) that seeks to institutionalize PPIUD services, both immediate and delayed insertion, as a routine part of antenatal counselling and delivery room services in Sri Lanka, Nepal and Tanzania.

## Methods

### Aims

This trial aims to determine the causal effect of the intervention on the uptake and subsequent continued use of PPIUD. The extent to which the intervention leads to the institutionalization of PPIUD services in the hospitals during and after the FIGO intervention, and to what extent the services diffuse to other hospitals or providers will also be assessed. In addition, the coverage and quality of postpartum contraceptive counselling and service provision will be measured, including women and providers’ satisfaction with these services.

Specifically, the study aims to determine:

1. The coverage and quality of counselling for postpartum family planning
2. The causal effect of the intervention on effective access to postpartum IUD during and 12 months after the end of the intervention period
3. The causal effect of the intervention on utilization of postpartum IUD during and 12 months after the end of the intervention period
4. The causal effect of the intervention on postpartum contraceptive use and method mix
5. The quality of the IUD services provided during the intervention period measured by expulsions, complications, and discontinuation
6. The causal effect of the intervention on postpartum reproductive behaviour and outcomes, including pregnancy, fertility, and abortion
7. The satisfaction of providers with the training they received, provision of services and their perspectives on scaling up PPIUD services
8. The satisfaction of women with postpartum IUD services and reasons for non-use and discontinuation
9. The institutionalization and diffusion of the PPIUD intervention
10. Health system and cultural facilitators and barriers to the successful implementation, institutionalization, and diffusion of postpartum IUD services

### Ethics approval

Ethical approval as exempt was granted by the Harvard T.H. Chan School of Public Health Office of Human Research Administration as Harvard will only receive de-identified datasets, and each country received ethical approval from the appropriate national Institutional Review Board (IRB). More specifically, the study received approval from the Ethics Review Committee at the Faculty of Medicine at the University of Colombo in Sri Lanka, the Nepal Health Research Council, and the National Institute for Medical Research in Tanzania. Protocol amendments were submitted for review and approval to each country’s IRB whenever changes to the protocol, consent forms, or questionnaires were made.

### Randomization of hospitals

Because of the potential benefit of the intervention to all women, a cluster-randomized stepped wedge design was considered most appropriate for the study. Six hospitals were selected in each country to provide national geographic coverage, and were randomized into two groups of three. Facilities were matched by number of deliveries per year, and one was randomly selected from each pair to be assigned to Group 1 while the other was assigned to Group 2. Using Stata software, researchers at Harvard T.H. Chan School of Public Health generated a random number that was assigned to each hospital. The hospital with the higher random number in the pair was assigned to Group 2, while the hospital with the lower random number in the pair was assigned to Group 1. A full list of study facilities is available upon request. The study enrolment period for both groups will be 12 months in Tanzania and 18 months in Nepal and Sri Lanka. In Group 1, the baseline standard of care (SOC) period will be three months and the PPIUD intervention period will be nine months in Tanzania and fifteen months in Nepal and Sri Lanka. In Group 2, the baseline (SOC) period will be nine months and the PPIUD intervention period three months in Tanzania and nine months in Nepal and Sri Lanka. We can represent this design for each group as follows:

**Table:** 

Time (months)	1-3	4-9	10-12 (Tanzania) 10-18 (Nepal and Sri Lanka)
Group 1 (Hospitals 1-3)	O	X	X
Group 2 (Hospitals 4-6)	O	O	X
where X = PPIUD intervention and O = control (standard of care provided)			

Due to the randomized nature, the causal effect of the intervention on the outcomes of interest can be estimated. In months 4-9, a randomized contrast can be made that will be controlled for any secular trends in outcomes. In addition, before-after contrasts will be made within hospitals controlled for all time-invariant differences in event rates between hospitals. The reference population for the study will be all women in the catchment area delivering in each hospital within a period of 12 months from the start of the study in Tanzania and within a period of 18 months in Nepal and Sri Lanka.

### Participant selection criteria

#### Women

Selection criteria for the study vary by country based on local regulations and the structure of the health system. In Sri Lanka, women will be eligible to participate in the study (inclusion criterion) if they deliver in one of the six study hospitals during the 18-month enrolment period, unless they normally reside outside of Sri Lanka. The study will interview women at up to four time points: baseline (in the hospital soon after delivery), 4-8 weeks postpartum for those who have PPIUD inserted, 9 months postpartum, and 18 months postpartum. In Sri Lanka, primary care services such as child immunizations are not available in the study hospitals, and the 9-month and 18-month surveys will be completed in satellite clinics affiliated with the study hospitals. There can be many satellite clinics affiliated with each hospital, and three to four will be selected based on geographic representation and the percentage of women delivering in the study hospitals who may receive postnatal care at the selected satellite clinics. Women enrolled at baseline will be eligible to complete the 9-month and 18-month surveys if they live in the catchment for one of the selected satellite clinics.

In Nepal, women will be eligible to participate in the study if they deliver in one of the six study hospitals during the 18-month enrolment period, unless they normally reside outside of the country. As in Sri Lanka, the study will interview women at up to four time points: baseline (in the hospital soon after delivery), 4-8 weeks postpartum for those who have PPIUD inserted, 9 months postpartum, and 18 months postpartum. In Nepal, because of the mountainous terrain and long travel times to reach remote areas, only women who live within 24 h of the study hospital will be eligible for the 9-month and 18-month follow up. Due to the high delivery caseload in the six hospitals selected in Nepal, a 55% sample of women enrolled at baseline who live within 24 h of the study hospital will be randomly selected to complete the two long-term follow up interviews at 9 months and 18 months postpartum. The long-term follow-up interviews will be conducted in the hospital when possible, but for participants unable to return to the hospital, enumerators will conduct interviews at the participant’s home or another location of the participant’s choosing.

In Tanzania, women will be eligible to participate in the study if they are age 18 or older and deliver in one of the study hospitals during the 12-month enrolment period, unless they normally reside outside of Tanzania. As in Sri Lanka, primary care services in Tanzania are not available in the study hospitals, and the 9-month and 18-month surveys will be completed in satellite clinics affiliated with the study hospitals. Three to four clinics will be selected based on geographic representation and the percentage of women delivering in the study hospitals who may receive postnatal care at the selected satellite clinics. In Tanzania, selection for the follow-up surveys is not dependent on enrolment at baseline. Data collectors will be posted at the selected satellite clinics, and women may be enrolled in the study at the time of the 9-month or 18-month survey when they visit the satellite clinics for child immunization services if they meet the eligibility criteria for the study (delivered in one of the six intervention hospitals during the 12-month enrolment period, normally reside in Tanzania, and are age 18 or older).

#### Providers

The provider sample includes all medical personnel who could potentially provide PPIUD who are working in the study hospitals during the 15-month intervention period, along with those who are working in the hospitals 12 months after the end of the study period. In Sri Lanka, only doctors are eligible to provide PPIUD services, and all eligible doctors were included. In Nepal and Tanzania, a random sample of providers was selected to participate in the Provider Survey. A full list of doctors and nurses eligible to provide PPIUD services was obtained, and stratified random sampling was used to select an equal number of providers from each cadre in each facility to complete the survey.

### The intervention

The International Federation of Gynaecology and Obstetrics (FIGO) has designed and will implement, through its nationally-affiliated Associations of Gynaecologists and Obstetricians, an intervention program on PPIUD services. As part of this Program, FIGO is responsible for the development and production of informational materials, training providers in counselling and in the provision of PPIUDs, improving facilities, ensuring good quality of service, and monitoring the program. Each component of the intervention is described in detail below.

#### Coordination with ministries of health

In order to introduce the provision of PPIUD services in a manner that is sustainable and achieves integration as part of the standard training and delivery practice, FIGO and their national affiliates will work closely with each country’s Ministry of Health to ensure adherence to national guidelines and to ensure quality of training and monitoring activities.

#### Training of providers

The facilities chosen in each country are primarily teaching hospitals who wish to include PPIUD as part of the regular OB/GYN training program and to integrate it as part of the standard delivery practice in these institutions. Health professionals will be trained through a combination of specific classroom-based training sessions and on the job training and mentoring, with new staff undertaking training as they rotate to the facilities.

Courses for approximately 12 master trainers, two from each participating hospital, will be organized. The training will include lectures, videos, web-based learning material, master classes and practical exercises on mannequins. Supervised IUD insertions and counselling sessions on patients will subsequently take place in order to establish competency.

These master trainers, who will be respected health care professionals with experience in implementing evidence-based clinical practices, will subsequently train the resident obstetrician/gynaecologists, the health facilities’ delivery team, the facilities’ antenatal team, and the community midwives in PPIUD related service delivery. All health providers involved in the project will be trained on counselling women on the benefits of adopting a postpartum contraceptive method for birth-spacing and maternal and infant health.

#### Training of community intermediaries

Community-based intermediaries, including midwives, skilled birth attendants and community health workers, are often the first point of contact for pregnant women in semi-urban areas. In an effort to increase the acceptance of PPIUD services, this intervention will orient community intermediaries linked with each hospital on postpartum contraception and PPIUD to enable them to integrate postpartum family planning content into their regular community-level activities, including counselling of pregnant women.

#### PPIUD service delivery

Women presenting at selected hospitals and associated clinics will receive information on postpartum contraception and the availability of PPIUD services during antenatal care and after delivery. The intervention will support printing of local government-approved leaflets on postpartum contraception and PPIUD to be used during counselling. In addition, the intervention will supply at least one TV for each hospital, and a video on postpartum contraception and PPIUD will be played in the local language. Informed consent for PPIUD will be obtained at the time of counselling and case notes marked for easy identification. Consent for PPIUD will be taken again immediately prior to PPIUD insertion and noted in the maternity records. Those women who have an IUD fitted will be encouraged to attend a follow-up visit with her midwife or the hospital/associated satellite clinic. Where possible, follow-up will be combined with the child’s 4-8-week immunization visit.

Each selected facility has a facility coordinator who will be responsible for working with the National Project Coordinator to have an effective stock management system in place and for ensuring that there are sufficient supplies of IUDs, proper inserters such as long curved Kelly’s forceps, clinical notes, patient information literature and consent forms.

#### Monitoring and mentoring

In order to track progress against the intervention’s objectives and to improve provider performance, FIGO and the in-country FIGO affiliates will regularly monitor service delivery data to provide support, if needed, to improve the performance of providers at the participating hospitals.

### Outcome measures

Study outcomes will be assessed using a variety of research instruments. Quantitative interviewer-administered surveys will be conducted with women at up to four time points:

1. Baseline (in hospital after delivery)
2. 4–8 weeks after delivery among women who accepted PPIUD
  - Providers ask women to get a PPIUD check-up at 4-6 weeks postpartum, and these data can be collected between 2-12 weeks after delivery
3. 9 months after delivery
  - Can be collected 8-14 months after delivery
4. 18 months after delivery
  - Can be collected 15-21 months after delivery

Quantitative interviewer-administered surveys will also be conducted with providers at three time points:

1. Baseline, prior to implementation of FIGO intervention
2. 6 months after implementation begins
3. 12 months after implementation ends

A facility survey using a checklist will also be completed before the intervention, 6 months after the intervention has begun, and 12 months after the end of intervention implementation to examine the extent to which PPIUD counselling and services have been institutionalized.

Primary and secondary outcomes are listed in Additional file 1, and key outcomes of interest are described in greater detail below:

1. Uptake of PPIUD
  To ascertain what percentage of women delivering in study hospitals take up PPIUD, participants will be interviewed after delivery, before they are discharged from the hospital
2. Receipt of PPIUD counseling before or after delivery
  To ascertain what percentage of women delivering in study hospitals receive counseling on PPIUD, participants will be interviewed after delivery, before they are discharged from the hospital. They will also be asked questions to assess the quality of the counseling.
3. PPIUD expulsion rate and complication rate at 4-8 weeks postpartum
  Participants who have not deliberately removed the PPIUD will be examined by providers at 4-8 weeks postpartum, and providers will report whether the PPIUD was expelled or complications occurred.
4. Modern contraceptive use at 9 months postpartum
  Participants will be interviewed at 9 months and asked whether they are using a modern method of contraception, defined as pill, implant, injectable, IUD, condom, or sterilization for the purposes of this study.
5. Modern contraceptive use at 18 months postpartum
  Participants will be interviewed at 18 months and asked whether they are using a modern method of contraception.
6. Pregnancy rate at 18 months postpartum
  Participants will be interviewed at 18 months and asked whether they are currently pregnant or have had a pregnancy that was terminated.
7. Sustainability: Percentage of trained providers who are still providing PPIUD services 12 months after the end of implementation
  Providers originally trained under the FIGO intervention will be interviewed at 12 months after intervention implementation ends to assess whether PPIUD services have continued after intervention support ends, suggesting institutionalization of services in the facility.
8. Sustainability: Percentage of trained providers providing PPIUD services in new facilities (after transfer) 12 months after the end of implementation
  It is expected that some providers originally trained under the FIGO intervention will be transferred to new facilities as is the practice in all three study countries. Providers originally trained under the FIGO intervention and transferred to new facilities will be interviewed 12 months after implementation ends to ascertain whether they continue providing services in their new facilities, diffusing PPIUD services to other parts of the country.
9. Sustainability: Percentage of new providers providing PPIUD services in intervention facilities 12 months after the end of implementation

New providers not originally trained under the PPIUD intervention, but who are working in intervention facilities will be interviewed 12 months after the end of intervention implementation to assess the diffusion of PPIUD services to other providers within the facility.

**Fig. 1:**
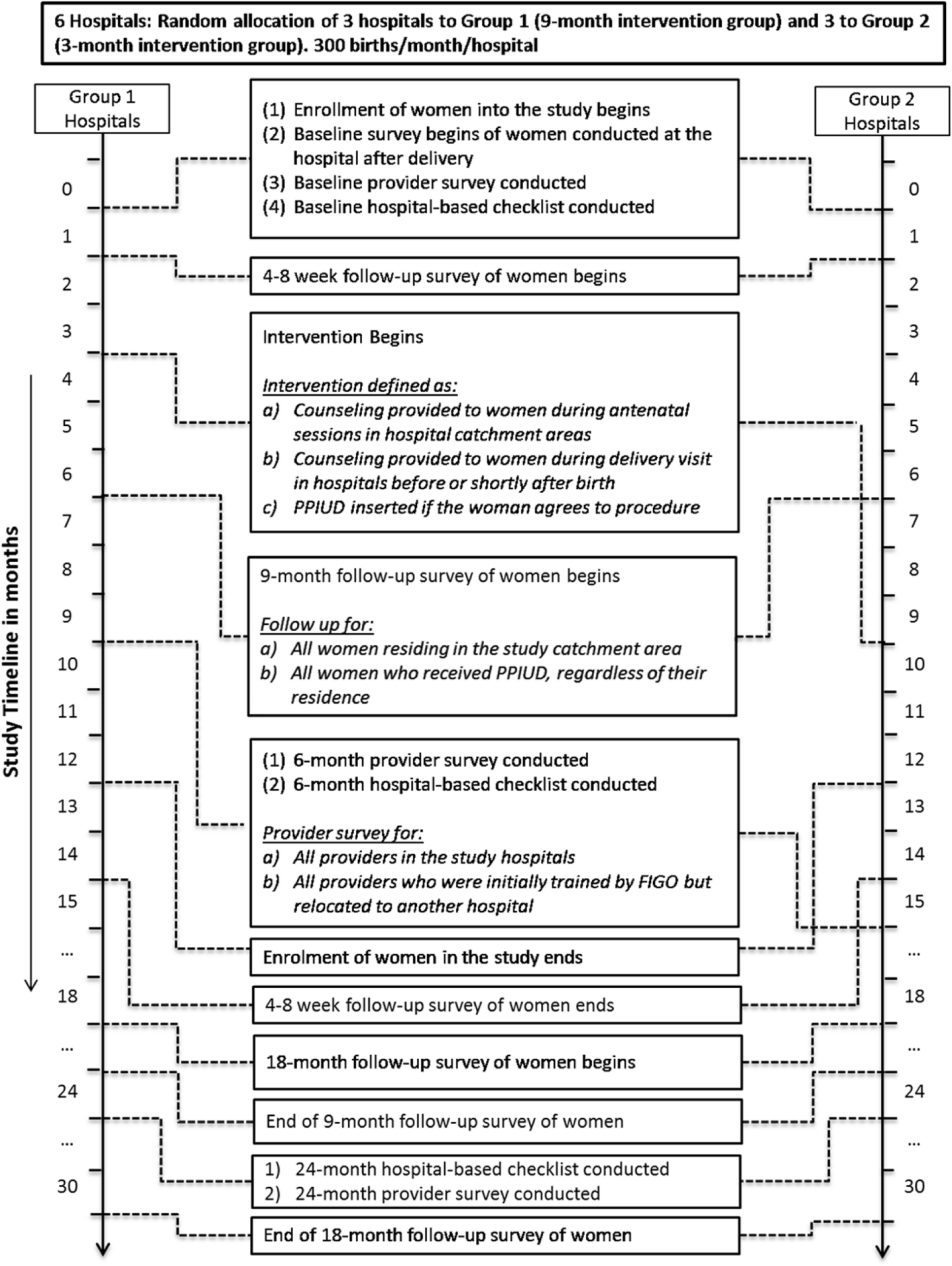
SPIRIT Flow Diagram for Tanzania: PPIUD study quantitative data collection by hospital group

**Fig. 2:**
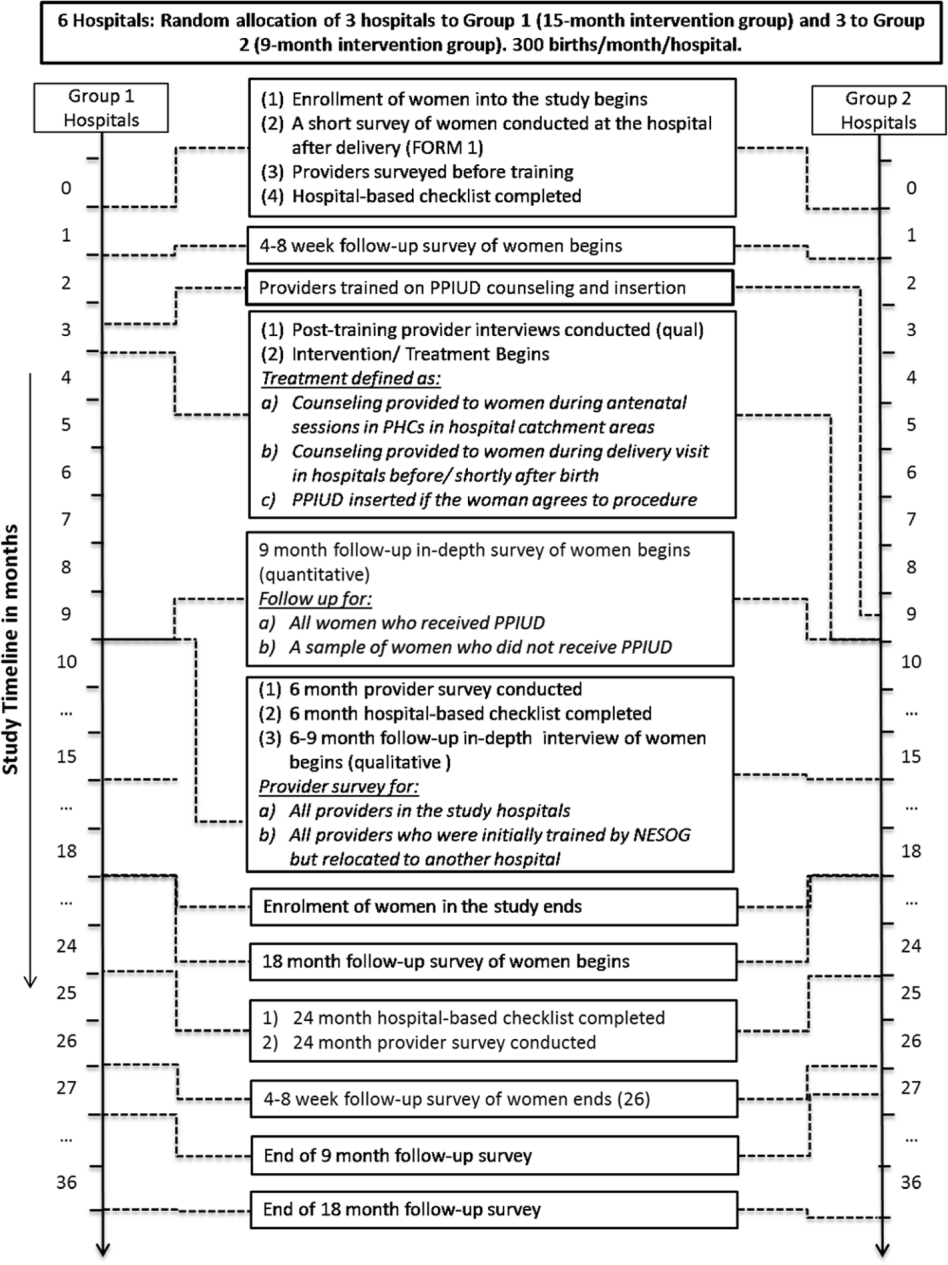
SPIRIT Flow Diagram for Nepal and Sri Lanka: PPIUD study quantitative data collection by hospital group

The study timeline and associated quantitative data collection activities for each of the two groups of hospitals are depicted in Fig. 1 for Tanzania and Fig. 2 for Nepal and Sri Lanka.

### Sample size

Following the cluster-randomized stepped-wedge design, power calculations were performed on a per country basis, assuming that 300 women are enrolled per hospital per month over a 12-month study enrolment period in Tanzania and an 18-month study enrolment period in Nepal and Sri Lanka, and followed for up to 18 months postpartum to ascertain study endpoints [13]. It is expected that the study will enrol 21,600 women in Tanzania and 32,400 women in Nepal and Sri Lanka.

1. PPIUD Counselling – Assuming that at the study hospitals during the standard of care time periods, the PPIUD counselling rate will be 1% or lower, we will have 80% power or more to detect an increased counselling rate of 2% or greater, even with an ICC of 15% [14] or lower, assuming that at least 21,600 women are enrolled during the 12-18-month enrolment period.
2. PPIUD Uptake – Assuming that at the study hospitals during the standard of care time periods, the PPIUD uptake rate will be 1% or lower, we will have 80% power or more to detect an increased PPIUD uptake rate of 8% or higher, even with an ICC of 15% or lower.
3. PPIUD Expulsion Rate at 6 weeks – Assuming that the PPIUD uptake rate increased to 8% during the intervention periods at the study hospitals, there will be 80% power or greater if the PPIUD expulsion rate is assumed to be 20% before the intervention [15] and drops to 5% or less after the intervention, for an ICC of 5% or less, even with 20% loss to follow-up postpartum or less. These analyses apply only to the intervention periods at the six hospitals, and are based upon a one-sample test for clustered data.
4. Modern contraceptive use at 6 weeks and 18 months – Assuming that modern contraceptive use at 6 weeks will be 55% in Sri Lanka [16] and that it will increase to 62% due to the availability of PPIUD during the intervention periods, there will be 80% power or more to detect this difference or anything greater, even if 20% of the participants enrolled during the antenatal period are lost to follow-up by the six week postpartum milestone and if the ICC is 15% or less. In Tanzania and Nepal, power is greater due to significantly lower modern contraceptive use at 6 weeks in the control group. Scenarios are similar for modern contraceptive use at 18 months.
5. Proportion pregnant within 18 months of index pregnancy – Assuming that 24% of women who enrol during the control periods are pregnant again within 18 months [17], there will be 80% power or more to detect a decreased proportion pregnant by 18 months of 19.4% or less, even if 20% of the clients enrolled during the antenatal period are lost to follow-up by 18 months and if the ICC is 15% or less.

Thus, we conservatively expect excellent power within each of the 3 countries for all primary endpoints of this study, assuming 21,600 in Tanzania and 32,400 women in Nepal and Sri Lanka are enrolled across the study hospitals at a rate of 300/month. If between-country heterogeneity analysis indicates that the effect sizes between the countries appear to be comparable, we will pool the results across the three countries for substantially greater power.

### Data collection procedures

#### Quantitative

All quantitative data will be collected by trained interviewers using an electronic Computer-Assisted Personal Interview (CAPI) format using the Dimagi CommCare survey management system on Android-based tablets. At baseline, women will be approached by study interviewers in postnatal wards and asked if they are interested in learning about the study. Women who are interested in learning more are provided with an informed consent form (Additional file 2) that is read aloud by the study interviewer. Women who consent to participate will sign or provide a thumbprint. The signature is captured electronically on the tablet, but women will be given the option to sign the paper copy if they prefer not to sign electronically. Tablets are used to take a photo of paper copies of signatures or thumbprints so that provision of consent is fully electronic. Baseline interviews collect information on the extent of counseling the woman received and to assess the primary outcome, PPIUD uptake. At the time of the baseline interview, women will be asked to provide their contact information, including their phone numbers as well as those of friends and family and directions to their homes to facilitate contact for follow-up interviews.

At the end of the baseline interview, women who have accepted PPIUD will be asked to return to the facility for a PPIUD check-up 4–6 weeks after delivery. Data collectors will complete the 4-6 week survey when participants return to the health facility for this check-up. The 9-month and 18-month surveys are based on the Demographic and Health Surveys (DHS) questionnaire format and will be conducted when women return to the health facility for their child’s vaccinations. Women who do not return to the health facility as scheduled will be called and asked to come to the facility for their interview. Women’s travel costs to the health facility will be reimbursed by the study. In the event that women are unable to return, interviewers may attempt to interview them at their homes or another convenient location. Data quality will be ensured through phone re-interviews of approximately 5% of study participants, conducted by the interview supervisor.

Providers selected for the baseline survey will be interviewed using the CommCare survey management system 6 months after the intervention begins, and 12 months after the intervention ends by trained interviewers. In the follow-up surveys, all providers who completed the baseline interview will be selected, including those who have left the facility, in order to capture the extent to which diffusion has occurred. At 12 months after the end of the intervention, new staff at the study hospitals, who arrived after the intervention period was completed, will also be sampled to evaluate the extent to which PPIUD has been institutionalized. Extensive follow-up information is collected on each provider’s contact information as well as information of a friend or family member to aid in retention.

#### Qualitative

Quantitative findings will be contextualized by qualitative in-depth interviews (IDIs) with providers and women to evaluate performance of the intervention. Twelve IDIs will be conducted with providers after they have received training through the FIGO intervention to understand their experiences with the training and facilitators as well as barriers to intervention implementation in their hospital. Two providers will be recruited from each hospital and purposively selected to represent different cadres of provider, where multiple cadres are trained, or by provider’s sex. Twenty-four IDIs will be conducted with women who have had at least two antenatal care visits following the start of the intervention to explore women’s experiences with postpartum contraceptive counselling during antenatal care and decision-making regarding postpartum contraceptive use. Four women will be purposively selected from each hospital based on socio -demographic characteristics. Finally, IDIs will be conducted with an additional 36 women who received a PPIUD after intervention implementation. These IDIs will be conducted at least nine months postpartum to understand women’s experiences using the PPIUD. Six women will be purposively selected from each hospital based on whether they have continued or discontinued PPIUD over the postpartum period. Interviews will be audio-recorded with participants’ permission, transcribed verbatim in the local language, and translated to English.

### Data management and monitoring

Data will be securely transferred from the Android tablets onto a CommCare-supported secure cloud server at the end of each working day. The CommCare cloud server is HIPAA-compliant and meets all the necessary security requirements for storing Level 4 identifiable data as defined by Harvard University. Once the data have been securely transferred to the cloud server, the survey record on the Android tablet is immediately erased. All data uploaded to the CommCare cloud server is encrypted and password-protected in accordance to the Level 4 data security and storage regulations. For each collected data case, which will consist of a participant data record, all personal identifiable data will be separated from the other non-identifiable data. The de-identified data will then be uploaded to an encrypted password-protected FTP site on a daily or weekly basis and will be circulated to the project investigators for analysis purposes for the duration of the study. Internal consistency checks and data validation are built into the CommCare software, but de-identified data are also cleaned monthly to promote data quality. Identifiable data will be stored separately from the de-identified data on the CommCare secure encrypted server for the duration of the study. Identifiable data will only be accessed by in-country collaborators for the purpose of revisiting participants at the appropriate follow-up periods.

Each country will have an independent Data and Safety Monitoring Board (DSMB) to be comprised of three to five members with at least: one epidemiologist or biostatistician, one reproductive health expert, preferably an obstetrician/gynaecologist (OB/GYN), and one social scientist or ethicist. Each DSMB will meet every four months to review interim results and monitor compliance with the study protocol and review any adverse events associated with the intervention, such as higher than expected expulsion or complication rates. Additional information can be found in the Terms of Reference for the PPIUD study DSMBs, which is available upon request.

Study enumerators are responsible for filling out a Protocol Deviation/Adverse Event Report. Reports will be submitted to the interview supervisors, and reviewed by the national study coordinator and the principal investigator. Reports will also be shared with the country’s DSMB and IRB.

### Planned analyses

#### Quantitative analyses

To assess the causal effect of the intervention on the antenatal counselling for PPIUD uptake, successful PPIUD insertion, and continued use of PPIUD 9 and 18 months following delivery, individual-level conditional logistic regression models will be used, stratifying on hospital, and controlling for calendar time to remove bias due to secular changes in outcomes unrelated to the intervention, with the causal effect estimated by a binary indicator of the status (standard of care or intervention) of each woman’s hospital at the time of her delivery. Semi-non-parametric methods to model secular trends will be used to control for confounding by secular trends as finely as needed [18].

Due to the randomized stepped-wedge design, results will be reported adjusted only for hospital and calendar time, alongside full multivariable adjusted results, in which all measured known or potential determinants of these outcomes are controlled for. Risk differences and risk ratios will be reported, as transformations of the odds ratios to be estimated by the conditional logistic regression model. Causal inference will be strongest when analysis is restricted to the subset of women enrolled during months 4-9 of the project, although power will likely be insufficient to detect effects of realistic sizes.

Selective loss to follow-up could be a concern for valid estimation of the causal effect of the intervention on PPIUD utilization 9 and 18-months post-delivery of the index pregnancy, but active follow-up with home visits will minimize this potential bias. Also, to account for this, marginal structural models for dependent censoring will be applied [19, 20], weighting individual observations by the inverse of their probability not to be lost to follow-up, as a richly parameterized function of all measured potential determinants of the relevant outcome. Attrition analysis would examine whether the characteristics of women lost to follow-up are different than those completing follow-up interviews. Effect modification by country and other key features will be explored by assessing the significance of any differences observed through likelihood ratio tests in which the relevant cross-product term is in and out of the model and by reporting stratum-specific point and interval causal effect estimates for any statistically significant differences. Should no effect modification by country be evident, we shall aggregate the data from the three countries and produce a single summary effect estimate as above. Standard meta-analysis techniques will be used to assess between country differences, including the Q-test [21], I^2^ [22] and CV_*B*_ [23]. The study is not designed or powered to estimate differential effects by subgroups of providers, facilities, or women, and such analyses will be undertaken with the caveat that it is suggestive rather than causal.

In additional secondary analysis, at the hospital level, we will employ a regression discontinuity approach by comparing women who deliver in the hospital immediately prior to the intervention with women who deliver immediately post intervention to obtain an estimate of the causal effects of the intervention on the outcomes of interest in each hospital.

The analysis of the provider and facility surveys will be undertaken to estimate the extent of institutionalization and diffusion. We will estimate to what extent providers continue providing the service when they move to other institutions. We will examine these effects for staff at different levels of the provider workforce.

#### Nested mixed-methods performance evaluation

The qualitative analyses that are part of this study will be closely integrated in the design, implementation and analytical stages of the quantitative component. Qualitative and quantitative data collection will be carried out synchronously. Analyses of the quantitative and qualitative data will initially be conducted separately, and then integrated later through iterative comparison of results and their relationships using a mixed methods approach.

### Dissemination

Trial results will be published in peer-reviewed journals, and presented at national and international meetings and conferences. Authorship will be determined by a publications committee, which includes members from all organizations involved in the study. Briefs indicating policy and programmatic implications of findings will be produced, and sessions will be organized involving national OB/GYN societies and government officials in discussions on translating evidence into action. Results are expected to guide the development of interventions to meet women’s need for unintended pregnancy prevention and enable policy makers and other stakeholders to make informed decisions in supporting programs and policies. The evidence generated by the study will also contribute to developing or updating guidelines for postpartum contraception.

#### Availability of data and materials

To allow for utilization of study data for additional research, the de-identified dataset will be made publicly available following publication of trial results.

### Roles and responsibilities

The faculty leaders of the project undertake essential supervision activities. The key responsibilities of the designated full-time project principal investigator and faculty co-principal investigator include:

- Working with FIGO to mesh the research and evaluation with the intervention
- Undertaking visits to the three countries to provide technical advice and guidance
- Working with the National Societies of Obstetricians and Gynaecologists in each of the three countries
- Ensuring the quality of the survey and qualitative work in each country
- Reviewing adequate enrolment of study participants according to the project timeline
- Providing technical advice and supporting the work of other project team members
- Leading interim and final analysis of the data generated
- Disseminating information and lessons learned on postpartum contraception and PPIUD in particular during international, regional and national scientific conferences/meetings.

Other Harvard T.H. Chan School of Public Health faculty serve as co-investigators. The principal investigators and supervising faculty are supported by a postdoctoral research fellow, three half-time research assistants and a project manager.

At the national level, a full time country coordinator, employed by the field implementing partner institution, will assume overall responsibility for project activities, including its local financial management. The core functions of the National Coordinator will include:

- Organizing training activities and supervision of national project staff
- Putting in place the required data collection systems
- Ensuring that the quality of the data collected is maintained at a high level
- Reviewing the performance of the research staff at each participating hospital

## Discussion

The provision of PPIUD is critical for meeting women’s need for long-term but reversible contraceptive protection. This cluster-randomized stepped-wedge trial will assess the causal effect of FIGO’s intervention to increase PPIUD uptake and continuation, as well as to institutionalize and diffuse these services after intervention implementation ends, providing essential information for program implementers and policy makers. This is the first study of its kind with potential to yield results both on the effectiveness of the intervention to enable women to space their births, and on the effectiveness of the intervention approach to create sustainable access to PPIUD services.

## Additional files

Additional file 1: WHO Trial Registration Data. Information required by the World Health Organization on trial registration. (DOCX 16 kb)
Additional file 2: Example recruitment scripts and informed consent forms from Nepal. Recruitment script and informed consent form for women and providers in Nepal. (DOCX 29 kb)

## Abbreviations

CAPI: Computer-assisted personal interview
DHS: Demographic and health surveys
DSMB: Data and safety monitoring board
FIGO: Federation of gynecology and obstetrics
IDI: In-depth interview
IRB: Institutional review board
IUD: Intrauterine device
OB/GYN: Obstetrician/gynaecologist
PPIUD: Postpartum intrauterine device
SOC: Standard of care
