# Supplementary material for: Institutionalizing postpartum intrauterine device (IUD) services in Sri Lanka, Tanzania, and Nepal: study protocol for a cluster-randomized stepped-wedge trial
Source: BMC Pregnancy Childbirth. 2016 Nov 21;16:362. doi: 10.1186/s12884-016-1160-0 (PMC5117577; doi:10.1186/s12884-016-1160-0)
Supplement: Additional file 1: — WHO Trial Registration Data. Information required by the World Health Organization on trial registration. (DOCX 16 kb) [file 12884_2016_1160_MOESM1_ESM.docx]

# Appendix I. WHO Trial Registration Data

| Primary registry and trial identifying number | ClinicalTrials.gov NCT02718222 |
| --- | --- |
| Date of registration in primary registry | March 11, 2016 |
| Secondary identifying numbers |  |
| Source(s) of monetary or material support | Susan Thompson Buffett Foundation |
| Primary sponsor | Harvard T.H. Chan School of Public Health |
| Secondary sponsor | None |
| Contact for public queries | Iqbal Shah; [ishah@hsph.harvard.edu](mailto:ishah@hsph.harvard.edu) |
| Contact for scientific queries | Iqbal Shah; [ishah@hsph.harvard.edu](mailto:ishah@hsph.harvard.edu) |
| Public title | Studying the impact and performance of institutionalizing postpartum IUD services in Sri Lanka, Tanzania, and Nepal: Study protocol for a longitudinal cluster-randomized stepped wedge trial |
| Scientific title | Studying the impact and performance of institutionalizing postpartum IUD services in Sri Lanka, Tanzania, and Nepal: Study protocol for a longitudinal cluster-randomized stepped wedge trial |
| Countries of recruitment | Sri Lanka, Nepal, Tanzania |
| Health condition(s) or problem(s) studied | Postpartum contraception; birth spacing |
| Intervention | Postpartum Intrauterine Device (PPIUD) |
| Key inclusion and exclusion criteria | Ages eligible for study: 18 years and older in Tanzania; any age in Sri Lanka and Nepal Inclusion criteria: recently delivered in study hospital; live in country where delivered |
| Study type | Cluster-randomized stepped wedge trial |
| Date of first enrolment | September 7, 2015 |
| Target sample size | 86,400 |
| Recruitment status | Recruiting |
| Primary outcomes | Percentage uptake of PPIUD |
| Key secondary outcomes | Percentage of women receiving PPIUD counseling Percentage of PPIUD acceptors who have expulsions at 4-8 weeks postpartum Percentage of women using modern contraception 9 months postpartum Percentage of women using modern contraception 18 months postpartum Percentage of women pregnant at 18 months postpartum Percentage of trained providers who are still providing PPIUD services 12 months after the end of implementation Percentage of trained providers providing PPIUD services in new facilities (after transfer) 12 months after the end of implementation Percentage of new providers providing PPIUD services in intervention facilities 12 months after the end of implementation |
| Protocol date and version | November 2016; version 3 |
