# Supplementary material for: Institutionalizing postpartum intrauterine device (IUD) services in Sri Lanka, Tanzania, and Nepal: study protocol for a cluster-randomized stepped-wedge trial
Source: BMC Pregnancy Childbirth. 2016 Nov 21;16:362. doi: 10.1186/s12884-016-1160-0 (PMC5117577; doi:10.1186/s12884-016-1160-0)
Supplement: Additional file 2: — Example recruitment scripts and informed consent forms from Nepal. Recruitment script and informed consent form for women and providers in Nepal. (DOCX 29 kb) [file 12884_2016_1160_MOESM2_ESM.docx]

# Appendix II. Example recruitment scripts and informed consent forms from Nepal

**RECRUITMENT SCRIPT**

**WOMEN FORM 1**

**PPIUD STUDY – NEPAL**

***INTRODUCTION***

Hello. My name is _________________ and I am here on behalf of Center for Research on Environment Health & Population Activities (CREHPA), Nepal Society of Obstetricians and Gynaecologists (NESOG) and Harvard University. I would like to invite you to participate in a study in which we are trying to understand use of post-partum family planning and reproductive health services in Nepal. We are interested in trying to understand the experience of women who have recently given birth. Before we proceed, I would like to confirm your eligibility to be in the evaluation study by asking a few questions.

(**GO TO QUESTION 1a**)

***DETERMINING ELIGIBILITY***

| **No.** | **Question** | | **Response** | **Skip Rules** |
| --- | --- | --- | --- | --- |
| 1a | Are you a woman who has recently given birth? | | YES NO | IF NO, GO TO **END**  IF YES, GO TO **1b** |
| 1b | Do you live in Nepal? | | YES NO | IF NO, GO TO **END**  IF YES, GO TO **2a** |
| 2a | I would now like to give you some information so you can decide if you want to participate in the study. May I continue? | YES NO | | IF NO, GO TO **3**  IF YES, GO TO **2b** |
| 2b | Enumerator: Is this the first time you are speaking to the woman about consent? | YES NO | | IF YES/ NO: GO TO **INFOMED CONSENT FORM, PAGE 3** |
| 3 | Would it be possible for me to talk to you again about this later? IF YES: When? | YES NO | | IF NO, GO TO **END**  IF YES, **ENTER DATE AND TIME:** **DATE:**   \|  \|  \|  \|  \|  \|  \|  \|  \| \| --- \| --- \| --- \| --- \| --- \| --- \| --- \| --- \|   (**DD/MM/YYYY**)  **TIME:**   \|  \|  \| **:** \|  \|  \| \| --- \| --- \| --- \| --- \| --- \|   **AM / PM**  **SAVE AND EXIT FORM.** |
| **END** | Thank you for your time. I hope that you have a good day. |  | | **END VISIT.** |

**INFORMED CONSENT FORM**

**WOMEN FORM 1**

**PPIUD STUDY – NEPAL**

At this time, I would like to describe the study and your role as a participant. Once we have reviewed these details, I will ask to obtain your consent to participate in the form of a signature or a thumb print.

***Purpose of the study***

We are conducting a study on women’s health and family planning. I like to speak with you and to hear about your own experience, to know what you think, and to hear your own opinions. We are talking to about 51,000 women like you who availed the service from one of the six hospitals in Nepal.

***Procedures***

To understand your experience and opinions, we invite you to take part in this research study. If you accept, you will be required to talk with me. If you do not want to answer any of the questions then you are free to refuse, but it will be very useful for us if you try to answer as much as you can. Different people have different experiences and ideas and I want to hear what you yourself experienced or think.

The interview will take place within this facility that ensured your privacy and also convenient to you. The expected duration of the interview will be about 30-45 minutes.

***Benefits and incentives***

There will be no direct benefit to you but the information you provide is likely to help us to find out more about the contraceptive counselling services provided in this facility. If you are selected for a longer follow-up at 9 months and 18 months, we will compensate costs of travel to the facility for the follow-up visit. We hope that the results of the study will help to improve family planning services in the country in the future.

***Confidentiality***

All the information you provide will be treated in strict confidence and only for research purpose without any identifying information. The interview will not influence the services that you are entitled to in any way.

**Right to refuse or withdraw**

You are completely free to take part in this study or to refuse to do so. The choice is completely yours. Participation in this study is voluntary and you can choose not to answer any individual questions or all of the questions, if you don't like. Even after you agree to participate in the study, you will be free to leave the interview at any time you wish and/or to refuse to answer any question that you are uncomfortable with.

***Who has reviewed this project?***

This project has been reviewed by, and received ethics clearance through Nepal Health Research Council.

**Who to contact**

If you have a concern about any aspect of the study, you should ask to speak to the researchers who will do their best to answer your questions. You can contact National Principal Investigators Dr. Mahesh Puri on 5546487 during Monday to Friday 9:30am to 5:00 pm.

Any complaint about the way you have been treated during the study or any possible harm you might suffer will be addressed. Please contact Dr. Khem Karki, Member Secretary, Nepal Health Research Council, at 4254220, during weekdays during 10:00 am to 5:00 pm, Sunday to Friday.

1. **Do you have any questions about what I have just told you?**

YES [Interviewer, please answer any questions to the best of your ability]…….1

NO [Interviewer, go to the next question]…………………………………..….2

1. **Do you agree to take part in the study?**

YES [Interviewer, go to next question)] …………………………….………….1

NO [Interviewer, thank the interviewee and leave him/her]………………...…2

**Declaration of the Participant**

I have understood that the purpose of the study is to understand more about women’s health and family planning. I have read the above information, or it has been read out to me. I have had the opportunity to ask questions about it and any questions that I have asked have been answered to my satisfaction. I consent voluntarily to participate as a subject in this study and understand that I have the right to withdraw from the study at any time.

| **If agreed,** |  | |
| --- | --- | --- |
| Name of participant |  |  |
|  |  |  |
| Signature of participant |  | Date |
|  |  |  |
| Signature of person obtaining consent |  | Date |
|  |  | |
| Printed name of person obtaining consent |  |  |
|  |  |  |

**RECRUITMENT SCRIPT**

**PROVIDER SURVEY**

**PPIUD STUDY - NEPAL**

Hello! My name is _____________________. We are here on behalf of Center for Research on Environment, Health and Population Activities (CREHPA) and Nepal Society of Obstetricians and Gynaecologists (NESOG) to conduct a survey of health providers to study of the impact and performance of Institutionalizing Immediate Post-Partum IUD Services as a routine part of antenatal counselling and delivery room services in Nepal. I would like to invite you to participate in a study in which we are trying to understand use of post-partum family planning and reproductive health services in Nepal.

**(GO TO QUESTION 1a)**

| **No.** | **Question** | | **Response** | **Skip Rules** |
| --- | --- | --- | --- | --- |
| 1a | Do you work in this hospital? | | YES NO | IF NO, GO TO **END**  IF YES, GO TO **1b** |
| 1b | Do you provide vaginal or caesarean delivery services in this hospital? | | YES NO | IF NO, GO TO **END**  IF YES, GO TO **2** |
| 2 | I would now like to give you some information so you can decide if you want to participate in the study. May I continue? | YES NO | | IF NO, GO TO **3**  IF YES, GO TO **INFORMED CONSENT FORM** |
| 3 | Would it be possible for me to talk to you again about this later? IF YES: When? | YES NO | | IF NO, GO TO **END**  IF YES, **ENTER DATE AND TIME:** **DATE:**   \|  \|  \|  \|  \|  \|  \|  \|  \| \| --- \| --- \| --- \| --- \| --- \| --- \| --- \| --- \|   (**DD/MM/YYYY**)  **TIME:**   \|  \|  \| **:** \|  \|  \| \| --- \| --- \| --- \| --- \| --- \|   **AM / PM**  **SAVE AND EXIT FORM.** |
| **END** | Thank you for your time. I hope that you have a good day. |  | | **END VISIT.** |

**INFORMED CONSENT FORM**

**PROVIDER SURVEY**

**PPIUD STUDY - NEPAL**

**READ THE FOLLOWING CONSENT FORM**

**Now I will read a statement explaining the study.**

***Procedures***

Your facility is one of the six hospitals selected to participate in this study by CREHPA and NESOG. We will be asking you questions regarding your training and perspectives related to the provision of IUCD and particularly post-partum IUCD. Information collected about you and other providers during this study may be used by CREHPA/NESOG to discuss what improvements in Service provisions are needed and what additional research is needed.

The interview will take place within this facility that ensured your privacy and also convenient to you. The expected duration of the interview will be about an hour.

***Benefits and incentives***

There will be no direct benefit to you but the information you provide is likely to help us to find out more about the family planning services provided in this facility. We hope that the results of the study will help to improve family planning services in the country in the future.

***Confidentiality***

All the information you provide will be treated in strict confidence and only for research purpose without any identifying information. Neither your name nor the names of any other health workers who participate in this study will be included in any report. We are interested in knowing your views and perspectives.

***Right to refuse or withdraw***

You may refuse to answer any question or choose to stop the interview at any time. However, we hope you will answer the questions, which will benefit the services you provide and the nation. If there are questions for which someone else is the most appropriate person to provide the information, we would appreciate if you introduce us to that person to help us collect that information.

***Who has reviewed this project?***

This project has been reviewed by, and received ethics clearance through Nepal Health Research Council.

***Who to contact***

If you have a concern about any aspect of the study, you should ask to speak to the researchers who will do their best to answer your questions. You can contact National Principal Investigator Dr. Mahesh Puri on 5546487 during Monday to Friday 9:30am to 5:00 pm.

Any complaint about the way you have been treated during the study or any possible harm you might suffer will be addressed. Please contact Dr. Khem Karki, Member Secretary, Nepal Health Research Council, at 4254220, during weekdays during 10:00 am to 5:00 pm, Sunday to Friday.

1. **Do you have any questions about what I have just told you?**

YES [Interviewer, please answer any questions to the best of your ability]…….1

NO [Interviewer, go to the next question]…………………………………..….2

1. **Do you agree to take part in the study?**

YES [Interviewer, go to next question)] …………………………….………….1

NO [Interviewer, thank the interviewee and leave him/her]………………...…2

**Declaration of the Participant**

I have understood that the purpose of the study. I have read the above information, or it has been read out to me. I have had the opportunity to ask questions about it and any questions that I have asked have been answered to my satisfaction. I consent voluntarily to participate as a subject in this study and understand that I have the right to withdraw from the study at any time.

| **If agreed,** |  | |
| --- | --- | --- |
| Name of provider |  |  |
|  |  |  |
| Signature of provider |  | Date |
|  |  |  |
| Interviewer signature |  | Date |
|  |  | |
| Printed name of interviewer |  |  |
|  |  |  |
